# Health promoting hospitals: a study on educational hospitals of Isfahan, Iran

**DOI:** 10.15171/hpp.2016.04

**Published:** 2016-03-31

**Authors:** Atefeh Afshari, Firoozeh Mostafavi, Mahrokh Keshvari, Leila Ahmadi-Ghahnaviye, Maryam Piruzi, Elham Moazam, Kavak Hejab, Ahmad-Ali Eslami

**Affiliations:** ^1^Department of Health Education and Promotion, Isfahan University of Medical Sciences, Isfahan, Iran; ^2^Department of Community Health Nursing, Isfahan University of Medical Sciences, Isfahan, Iran; ^3^Seidolshohada Hospital, Isfahan University of Medical Sciences, Semirom, Iran

**Keywords:** Health promoting hospitals, Health promotion, Hospital

## Abstract

**Background: ** The current situation of health promotion (HP) services in hospitals of Iran is unclear. The aim of this study was to assess the status of HP in hospitals in Isfahan, Iran.

**Methods: ** This study is a cross-sectional survey in which 9 educational hospitals selected through census sampling. HP self-assessment was used for the data collection. The assessment teams formed, and evidence examined in line with the tools.

**Results:** The results identified five categories of HP activities in the hospitals consisted: patients,staff, environmental, community, and organizational. The mean of total score of HP was 48.8(9.8). In terms of the HP standards scores, 5 hospitals (55.5%) were at the intermediate level;3 hospitals (33.3%) were at the weak level, and 1 hospital (11.1%) was at the good level.About the standards, the highest score was "information and patient interventions" standard 79.8 (13.5), and the lowest was "continuity and cooperation" standard 36.2 (10.8).

**Conclusion:** It seems that some of the health promoting hospitals (HPS) duties carried out by hospitals. So, to improve the quality of health services, it seems useful to encourage policymakers and health service managers to create coherent policies and guidelines in HPS.

## Introduction

 Nowadays, the focus of hospitals just on treatment is criticized due to increased costs in health and chronic diseases caused by ageing population; thus, it is not surprising that health services modification, considering health promotion (HP) services and prevention of diseases are accentuated.^1,2^ Accordingly, with the presentation of setting-based HP strategies in Ottawa charter for HP, World Health Organization (WHO) suggests health promoting hospitals (HPH) as an effective strategy to modify health services.^3^ HPH is defined by WHO as follows:

 “A HPH does not only provides high quality and comprehensive medical and nursing services, but also develops a corporate identity that embraces the aims of HP, develops a health promoting organizational structure and culture including active and participatory roles for patients and all staffs, develops itself into a health promoting physical environment, and in brief, cooperates actively with community.”^1,4^

 At the beginning of the 1990s, the HPH and health services network was initiated by the WHO regional office for Europe to improve the quality of health care, increase relationship between hospitals/health services, community and environment as well as satisfaction of patients and staffs.^1,4,5^

 Since HP programs in hospitals are a part of the quality management programs for health services and HP standards have not also been regarded in health services quality standards, thus, WHO established a group to set new standards for HPH at the ninth International Conference on HPH - May 2001. After conducting pilot study and the final revision, 5 standards introduced:

1. “Patient assessment standard (S2): describes the organizations’ obligation to ensure the assessment of the patients’ needs for HP, disease prevention, and rehabilitation.”
2. “Patient information and intervention standard (S3): states that the organization must provide the patient with information on significant factors regarding to their disease or health condition and HP interventions must be established in all patients’ pathways.”
3. “Promoting a healthy workplace standard (S4): gives management the responsibility to establish conditions for developing hospital as a healthy workplace.”
4. “Continuity and cooperation standard (S5): deals with continuity and cooperation, demanding a planned approach to collaboration with other health service sectors and institution.”^6-8^

Since the establishment of HPH network, many progressions have occurred. Participation in this network was more prevalent in the Europe at the beginning, and today, it has included hospitals from other continents, such as Africa, Australia, and networks outside Europe, such as Montreal (2005), Taiwan (2006), and Toronto (2008) that are increasing in membership daily. Currently, this network consists of more than 900 registered hospitals and health care services in more than 40 countries.^1,9^

 Despite the progress in HP in hospitals in the European countries, this has not occurred in developing countries and has also been neglected in health care system of these countries.^10-18^ The current situation of HP services in hospitals of Iran is also unclear. Although some services, such as nutritional counseling and patient education are provided in some hospitals; however, there is no defined structure for many of these services, including smoking cessation services or HP services for asthma patients. The Iranian hospitals have different level of readiness and requirement to form a HPH; moreover, some of hospitals are not adequately equipped to achieve the HPH.^19^ Majority of the professionals (63%) believed that HP activities have not been provided in hospitals in Iran and 37% of these professionals suggested that these services are diffused and unorganized.^20^ According to the new approach of HPH in Iran, there are few studies and experiences and managers of treatment are recently attracted to this field. Evaluation of the HP standards in hospitals is the initial step to achieve HPH in the country. Therefore, the current study (which is a part of the doctoral dissertation in the field of health education and promotion with the aim of identifying the effective factors and processes in the adoption of HP in hospitals) reports the status of HP in hospitals in Isfahan, Iran. The results of this study, with making clear understanding of the status of HP in hospitals, can help hospitals move to the HPH.

## Materials and Methods

###  Participants and procedures

 A cross-sectional study was conducted at therapeutic educational hospitals in Isfahan city from March to June 2015. The target hospitals were therapeutic educational centers, and inclusion criteria were hospital official’s satisfaction for taking part in the study. Census sampling method was used, and nine hospitals participated in the study finally. To cooperate with hospitals, necessary permits were obtained and officials and staff of accreditation and health quality improvement in hospitals were invited after asking the manager of the accreditation unit of Treatment Administration of Isfahan University of Medical Science. In total, 9 hospitals out of the 11 hospitals that accepted invitation attended with accreditation manager in a meeting at department of health. The evaluation purposes and standards of HP in hospitals were introduced in the meeting, and the officials were persuaded to participate. The assessment team was finally formed after coordination with director of accreditation unit in the hospitals. In addition to the researcher, the assessment team included two experts in the accreditation unit of Treatment Administration who volunteered to work with researcher for hospitals assessment. Based on the nature of standards, the assessment team assessed each of the standards with the officials in the hospitals consisting of director or connector of accreditation, director or connector of quality improvement, educational supervisor, director or connector of patient and family education, occupational, and environmental health expert and social worker.

 In order to ensure the accuracy of the data, standard tools were used; all the assessment teams were trained to use tools, and several methods were used to data collection and examine the evidence for each standard (including interviews, observation, and documents review). For the validity of data, hospital officials assured the names of hospitals and results would be confidential and a code was assigned to each hospital, and grading objective was not considered for hospitals. However, the officials were willing to have a clear understanding of their HP situation.

###  Measures 

 The self-assessment form of HPH developed by WHO was used as the study tool.^21^ In Groene et al^22^ study, internal consistency was reported using Cronbach alpha coefficient as 0.77 to 0.88. In Yaghoubi and Javadi^23^ survey conducted in Iran, internal consistency was 0.78. In the present study, internal consistency 0.76-0.84 was calculated using Cronbach alpha for tool’s standards at 9 evaluated hospitals. These tools measure 5 standards including “management policy,” “patient’s assessment,” “information and intervention service,” “healthy workplace,” and “continuity and cooperation” at hospitals. Each standard involved 4 to 6 substandards, and there were 24 substandards to survey. In total, 68 measurable items were evaluated as yes (2 points), no (no points), and to some degree (1 point).

 “Policy management” standard was evaluated by the 17 measurable items scored 0 to 34. Some of the evidences examined in line with the aforementioned standard includes hospitals mission statement, strategic planning, project participation agreement with HPH, action plan and program quality improvement plan, audit activities, evidence of funds, implementation of clinical guidelines or patterns clinical segments, list of staff members to coordinate HP, educational pamphlets about HP policies, HP program, randomized interviews with employees, job duties, and HP facilities.

 The “patient’s assessment” and “information and intervention service” standards were measured using 8 items scored 0 to 16. Some of the explored evidences in these 2 standards include patients need assessment guideline, randomized study of patients’ medical records, randomized interview with personnel, evaluation of patients training satisfaction, public health education and high risk disease pamphlets in the printed or electronic copy form, and educational booklets of patients’ organizations.

 “Healthy workplace” standard was evaluated using 16 measurable items scored between 0 and 32. Some of explored evidences included in this standard were randomized review of personnel files, minutes of employee participation in hospital committees, personnel occupational and physical health records, randomized interview with personnel, presence of smoking cessation program, food menu of restaurant, healthy food availability policy, plan existence, and documentations of workplace risks evaluation.

 “Continuity and cooperation” standard was evaluated using 19 measurable items scored 0 to 38. Some of the reviewed evidences in this standard include interviews with members of the executive committee, partners collaboration documents, randomized check of medical records, post discharge guidelines, the list of collaborating organizations and partners, and comprehensive guideline about the exchange of information between organizations after discharge.

 General score for standards (overall score of HP at hospitals) ranged from 0 to 136. Since each substandard and standard with a different number of elements were evaluated, the score range (0 to 100) was converted in this study using proportionality instead.

 To clear describe understanding of HP situation and type of activities in the hospitals, based on self-assessment tool, observations and action plan of HP in hospitals were also considered.

###  Statistical analysis of data

 Obtained scores analyzed in SPSS 16 using descriptive statistics (mean, standard deviation [SD], median, and interquartile range [IQR]). Based on HP score, hospitals were categorized into 3 groups (weak, intermediate, and good) with cut point 2.

## Results

###  Hospital characteristics

 Assessed hospitals were educational with average of 261 (196.6) beds. Most hospitals had fewer than 200 beds. All hospitals (100%) were governmental, in which 3 (33.3%) were general and 6 (66.6%) were specialized. All hospitals had patient and family education unit, but only one hospital (11.1%) had HP unit. None of the hospitals were at HPH network. Table 1 shows the characteristics of the studied hospitals.

**Table 1:** Characteristics of Isfahan educational hospitals

**Variable**	**Number (%)**
Status of hospitals	
Governmental	9 (100)
Non-governmental	0 (0)
General	3 (33.3)
Specialized	6 (66.6)
Size	
≤200 beds	5 (55.5)
200-399 beds	3 (33.3)
400-599 beds	0
>599 beds	1 (11.1)
Health promotion & education unit status	
Patient and family education unit	9 (100)
Health promotion unit	1 (11.1)
Membership in HPH network	0

Abbreviation: HPS, health promoting hospitals.

###  HP standards in the hospitals

 According to the converted number of 0 to 100, the overall mean score of HP at the hospitals was 48.8 (9.8) and median was 47 (16.9). The lowest score was 34.5 and the highest was 63.2. In The HP standards scores, 5 hospitals (55.5%) were at intermediate level (cut point 43 to 59), 3 hospitals (33.3%) were at weak level (cut point ≤42), and just 1 hospital (11.1%) was at good level (cut point ≤60). Table 2 shows the frequency distribution of Isfahan educational hospitals levels based on HP score. Among the evaluated HPH standards, the highest score was for the S3 (Mean [SD] = 79.8 [13.5], Median [IQR] = 87.5 [15.6]). After that S4 (Mean [SD] = 56.2 [12.5], Median [IQR] = 56.2 [17.2]), and S2 (Mean [SD] = 52.8 [16.2], Median [IQR] = 62.5 [21.9]) found in the next grads, respectively. The lowest scores earned in S5 (Mean [SD] = 36.2 [10.8], Median [IQR] = 36.8 [13.7]) and after that in S1 (Mean [SD] = 39.2 [11.4], Median [IQR] = 35.3 [20.6]).

**Table 2:** Frequency distribution of Isfahan educational hospitals levels based on health promotion score

**Hospitals Level**	**I (Weak)**	**II (Intermediate)**	**III (Good)**
Cut points	≤ 42	43 to 59	≤ 60
N (%)	3 (33.3)	5 (55.5)	1 (11.1)

 In S1, the lowest mean score was 20.6 and the highest mean score was 52.9. Among evaluated sub-standards in S1, the highest score earned in S1.1 (Mean [SD] =75.5 [10.1], Median [IQR] =80 [5]). After that S1.6 (Mean [SD] = 38.8 [48.6], Median [IQR] = 50 [100]), S1.5 (Mean [SD] = 33.3 [12.5]), Median [IQR] = 25 [25]), and S1.4 (Mean [SD] =27.7 [23.1]), Median [IQR] =25 [50]) and S1.2 (Mean [SD] =27.7 [16.6], Median (IQR] =16.6 [33.3]) found in the next grads, respectively. The lowest score earned in S1.3 ([Mean [SD] = 11.1 [13.1]), Median [IQR] = 25 [25]).

 All the hospitals (100%) had aim, mission, and quality management plans include HP. Most of the hospitals (77.7%) had HP plan in the fields of patients, staff, community, and environment. However, there were no an identifiable budget for the HP plan unless for patient education in some hospitals (44.4%). Except managerial staff working in accreditation ward of hospital, none of the managers and health professionals were aware of the HP programs and policy. There were no introductory trainings, including HP for new staff. All the hospitals had partially a training program on HP attended for all staff, such as patient education, communication skills, infection control, etc. Most of the hospitals (77.7%) had not been necessary structures and facilities to carry out HP.

 The lowest and highest mean score in S2 were 18.7 and 68.7, respectively. In this standard, the highest score related to substandard S2.1 (Mean [SD] = 72.2 [36.3], Median [IQR] = 100 [50]). S2.3 (Mean [SD] = 59.2 [16.9]), Median [IQR] = 66.6 [8.3]) and then S2.2 (Mean [SD] = 50 [35.3]), Median [IQR] = 50 [50]) and S2.4 (Mean [SD] = 50 [0]), Median [IQR] = 50 [0]) found in the next grads, respectively. The lowest score related to S2.5 (score was zero).

 To identification of socio-cultural characteristics of patients and risk factors (such as smoking, alcohol consumption, nutritional status, disease family history, etc.), an early patient assessment form was attached in the patient’s medical and nursing records (88.8%). However, these forms documented partially and not used in the patients’ healthcare. In 77.7% of patient education units, educational needs of five prevalent patients group were identified (for example, asthma patients, diabetes patients, cardiovascular disease, surgery, etc.). In all of hospitals, detecting and documenting of patient risk factors were being performed by nurses only.

 In S3, the lowest mean score was 50 and the highest mean score was 93.7 and among the evaluated sub-standards, score (100) earned in S3.1 and S3.3. After that S3.5 (Mean [SD] = 85.2 [19.4], Median [IQR] = 100 [33.3]), and S3.2 (Mean [SD] = 72.2 [44.1], Median [IQR] = 100 [75]) found in the next grads, respectively. At least the lowest score earned in S3.4 (Mean [SD] = 55.5 [24.3], Median [IQR] = 50 [25]).

 In all hospitals, education and information services were being provided for patient and their family with focus on current disease (such as treatment, procedures, follow up times, nutrition, and home care) and were being documented in patient education forms. General health information on prevalence disease and patients organizations was available in hospitals (77.7%). Evaluating the effectiveness of educations was done partially in 66.6% hospitals. In all of hospitals, the patient educations were being performed by nurses only.

 In S4, the lowest mean score was 34.3 and the highest mean score was 75. In substandards of S4, the highest score earned in substandard S4.3 (Mean [SD] = 72.2 ([26.3], Median [IQR] = 50 [50]) and S4.4 (Mean [SD] = 56.9 [15.4], Median [IQR] = 50 [31.2]), S4.1 (Mean [SD] = 55.5 [5.3], Median [IQR] = 60 [10]), and S4.2 (Mean [SD] = 53.7 [24.3], Median [IQR] = 50 [45.8]) found in the next grads, respectively.

 In all of hospitals, there was an annual staff training plan on the health issues, and the new staff have been received an induction training that not address the HP program exactly. In all hospitals risk management training courses were being held but none of the hospitals had training courses in HP skills, specifically. In all of hospitals, working conditions had not complied with national or regional directives and indicators completely. In 44.4% of hospitals, workplace risks were identified. In most hospitals (77.7%), the staff underwent an annual health check-up. In 44.4% of hospitals, staff had access to variations of healthy food in the canteen. Hospital employees’ representatives had been involved in hospital committees, and participative management existed in some hospitals (22.2%). Likeness, in all hospitals the members of managerial staff were being more often involved in the hospital policy-making than non-managerial staff. There were no programs for detection of smoking staff and smoking cessation in hospitals. Just in 22.2% hospitals, there were educational pamphlets and brochures.

 The lowest and highest mean score in S5 were 21 and 52.6, respectively. Among substandards of S5, S5.3 had the highest score (Mean [SD] = 59.5 [10.1], Median [IQR] = 64.3 [17.8]), S5.1 (Mean [SD] = 29.1 [16.5], Median [IQR] = 25 [18.7]), and S5.2 (Mean [SD] = 21.3 [13.2], Median [IQR] = 16.6 [20.8]) found in the next grads, respectively. In this standard, the lowest score earned in the substandard S5.4 (Mean [SD] = 13.8 [13.1], Median [IQR] = 25 [25]). Table 3 shows the mean score of HP standards and substandards in the hospitals.

**Table 3:** The mean and median of total score and scores of health promotion standards and substandards in the Isfahan educational hospitals

**Standards and substandards**	**Min-Max**	**Mean (SD)**	**Median (IQR)**
Standard 1: management policy (N=17)	20.6-52.9	39.2 (11.4)	35.3 (20.6)
1-1 The organization identifies responsibilities for the process of implementation, evaluation and regular review of the policy (N=5)	50-80	75.5 (10.1)	80 (5)
1-2 The organization allocates resources to the processes of implementation, evaluation and regular review of the policy (N=3)	16.5-50	27.7 (16.6)	16.6 (33.3)
1-3 Staff are aware of the health promotion policy and it is included in induction programs for new staff (N=4)	0-25	11.1 (13.1)	25 (25)
1-4 The availability of procedures for collection and evaluation of data in order to monitor the quality of health promotion activities (N=2)	0-50	27.7 (23.1)	25 (50)
1-5 The organization ensures that staff have relevant competences to perform health promotion activities and supports the acquisition of further competences as required (N=2)	25-50	33.3 (12.5)	25 (25)
1-6 The organization ensures the availability of the necessary infrastructure in order to implement health promotion activities (N=1)	0‏-100	38.8 (48.6)	50 (100)
Standard 2: patient assessment N=8)	18.7-68.7	52.8 (16.2)	62.5 (21.9)
2-1 The organization ensures the availability of procedures for all patients to assess their need for health promotion (N=2)	0-100	72.2 (36.3)	100 (50)
2-2 The organization ensures procedures to assess specific needs for health promotion for diagnosis-related patient-groups (N=1)	0-100	50(35.3)	50 (50)
2-3 The assessment of a patient's need for health promotion is done at first contact with the hospital (N=3)	16.6-66.6	59.2 (16.9)	66.6 (8.3)
2-4 The patients' needs assessment ensures awareness of and sensitivity to social and cultural background (N=1)	50-50	50 (0)	50 (0)
2-5 Information provided by other health service partners is used in the identification of patient needs (N=1)	0	0	0
Standard 3: Patient information & intervention(N=8)	50-93.7	79.8 (13.5)	87.5 (15.6)
3-1 Based on the health promotion needs assessment, the patient is informed of factors impacting on their health and, in partnership with the patient, a plan for relevant activities for health promotion is agreed (N=1)	100-100	100 (0)	100 (0)
3-2 Patients are given clear, understandable and appropriate information about their actual condition, treatment, care and factors influencing their health (N=1)	0-100	72.2 (44.1)	100 (75)
3-3 The organization ensures that health promotion is systematically offered to all patients based on assessed needs (N=1)	100-100	100 (0)	100 (0)
3-4 The organization ensures that information given to the patient, and health promoting activities are documented and evaluated, including whether expected and planned results have been achieved (N=2)	0-75	55.5 (24.3)	50 (25)
3-5 The organization ensures that all patients, staff and visitors have access to general information on factors influencing health (N=3)	50-100	85.2 (19.4)	100 (33.3)
Standard 4: Promoting a healthy workplace (N=16)	34.3 -75	56.2 (12.5)	56.2 (17.2)
4-1 The organization ensures the establishment and implementation of a comprehensive human resources strategy that includes the development and training of staff in health promotion skills (N=5)	50-60	55.5 (5.3)	60 (10)
4-2 The organization ensures the establishment and implementation of a policy for a healthy and safe workplace providing occupational health services for staff (N=6)	16.6-83.3	53.7 (24.3)	50 (45.8)
4-3 The organization ensures the involvement of staff in decisions impacting on the staff's working environment (N=1)	50-100	72.2 (26.3)	50 (50)
4-4 The organization ensures availability of procedures to develop and maintain staff awareness on health issues (N=4)	37.5-75	56.9 (15.4)	50 (31.2)
Standard 5: Continuity & cooperation (N=19)	21-52.6	36.2 (10.8)	36.8 (13.7)
5-1 The organization ensures that health promotion services are coherent with current provisions and health plans (N=4)	12.5-62.5	29.1 (16.5)	25 (18.7)
5-2 The organization identifies and cooperates with existing health and social care providers and related organizations and groups in the community (N=6)	0-41.6	21.3 (13.2)	16.6 (20.8)
5-3 The organization ensures the availability and implementation of activities and procedures after patient discharge during the post-hospitalization period (N=7)	42.8-71.4	59.5 (10.1)	64.3 (17.8)
5-4 The organization ensures that documentation and patient information is communicated to the relevant recipient/follow-up partners in patient care and rehabilitation (N=2)	0-25	13.8 (13.1)	25 (25)
Hospital total score (N=68)	34.5-63.2	48.8 (9.8)	47 (16.9)

N=Number of item.

 There were no guidelines or procedures on how to cooperate with existing health and social care providers and related organizations and groups in the community. 33.3% of hospitals had limit and irregular partnership with Health Administration of Isfahan University of Medical Science. There were no written plans on how to refer patients to community health care organizations. However, in 77.7% of hospitals print information on community partners were provided (such as MS Association, Diabetic Clinic, Cancer Association, organizations for disabled people, etc.). There was discharge policy in all hospitals and follow up advice were recorded by the nurse in patient education forms and medical and nursing records.

###  HP and prevention programs in the hospitals 

 Since carrying out the accreditation program in the hospitals, all hospitals had annual HP and prevention program. The findings from assessing HP and prevention program identified four categories activities: patient, staff, environment, and community. The main focus of HP programs was on activities oriented toward patient and their family. This was through health education and information strategies. In the staff, the main focuses of activities were on occupational health and social, emotional, cultural, and recreational services. Activities oriented on the environment, related to the waste management program to improve recycling of medical and general waste. The lowest focuses of HP programs were on activities oriented toward community and, in three hospitals, there were no plans for community. However, the focus of activities was on public education and information strategies through telephone and mass media (the hospital website) and print media (in the hospital waiting room).

 The findings of the study also identified another category of HP activities as “organizational.” These activities were: the infection control program, occupational and patient safety program, risk management program, and adding units for patient education, HP (in one hospital), infection control, environmental health, and occupational health (in four hospitals). Activities in this area and environmental area tended to come under the banner of the quality management program (as part of the accreditation program), rather than being described as HP activities.

## Discussion

 HPH concept in Iran is new and until the date, there have been any legislative support and specific policy from the Ministry of Health and Treatment to developed hospitals to HPH. However, the hospital’s reform in Iran has been initiated focusing on reorganization of the hospitals with improving the quality of health services provided by hospitals. The findings of the study showed that HP program was not a priority in the hospitals and just considered as a part of the hospital accreditation programs. However, in line with the accreditation program; the hospitals engaged in HP activities that categorized in the fields of the patients, the staff, the environment, the community, and the organizational. These fields of HP considered in pilot projects of HPH and have been confirmed in other studies.^4,17,24,25^ Also based on HP standards in hospital, the mean score of HP at hospitals was 48.8 (9.8) and most of the hospitals were at intermediate level. In a similar study, Yaghoubi and Javadi^23^ reported the HP score as 51.3 (14.1) at governmental hospitals, that is, close to the present study, and 56.9 (16.1) at private hospitals, and this score was significantly higher in private hospitals than governmental hospitals. It seems that some of the HP activities are carrying out at hospitals of Iran and this can be a strong point to move HPH.

 The main focus of HP activities in the studied hospitals was on patient information and intervention. The results of Yaghoubi and Javadi study in Iran and other study were in line with the present study.^23,25-27^ Findings show there are strong education and information for patients and families at the hospitals and patient receive information about treatment, care, and factors affecting their health. However, effectiveness of educations is not systematically evaluated. It’s maybe related to lack of collaboration with other health professionals in patient education, limited time of nurses, and patient and work overload. So in line with Johansson et al study, it is necessary to improve the skills of hospital staff in evaluation of educational plan.^26,28^

 The lowest focuses of HP activities at the hospitals were on the community field and “cooperation and continuity” standard. Although the studied hospitals had some activities for the post-hospitalization period of patients, but they had limited role in HP of community and cooperation with other organizations and groups in the community. This challenge has been demonstrated in other studies.^23,26,27,29^ As Yaghoubi and Javadi^23^ study, in the present study, there were lack of guidelines for cooperation and exchange information with other organizations in community. Johnson and Nolan also highlight the lack of a mechanism for hospital staff at all levels to meet and establish networks with community organizations both formally and informally.^29^ Thus, it is necessary to discuss about this issue in hospital management guidelines and specify a clear plan for post discharge and guideline for cooperation and information exchange with organizations.

 After S5, hospitals gained low score in S1. The objective of this standard is to describe the framework for the organization’s activities concerning HP. To achieve an HPH, it is vital to consider HP in hospital mission, goals and values, vision, strategies, and policies must be developed to direct activities.^30,31^ Despite the HP mission in the hospitals and carrying out a series of HP activities, HP was not seen as a priority policy in hospitals. As there was low-level staff awareness of the HP programs, lack of training programs, and lack of funding and facilities for HP activities. A case study in Sweden also has documented that, in reality, leaders might not consider preventive and health-promoting efforts to be a first priority.^32^ In the Iran, this might be related to low priority of health prevention and promotion in the health system and treatment-centered organizational culture in hospitals. To move toward HPH, it is necessary to create various national policies and regulations that built a supportive context to implement of HP.

 The result shows mean score of the S2 and S4 close to each other. In Yaghoubi and Javadi study, the score of S2 was lower than that in the present study 44.2 (20.1), and reported “hospitals did some assessments for patients; however, they did not consider the socio-cultural aspects.”^23^ But in present study, exploring documents showed that socio-cultural characteristics of patients listed and recorded in the early assessment and patient training forms. This conflict maybe related to the time of evaluation. In line with accreditation program, performing the patient assessment measures have started gradually in 2013 and maybe performing this standard has improved overtime. However, the results showed that as S3, patient assessment is performed by the nurses only. Furthermore, the assessment results are used less in nursing or physician interventions and in patients referring time. It is maybe related to lack of health professional team working, lack of staff training, and lack of clear guidelines.

 Hospital staff is at risk of a great number of hazards at workplace, including biological, chemical, physical, ergonomic, and psychosocial risks.^33^ S4 refers to create healthy working environment for staff. In most sustandards, the earned scores were somewhat similar and S4.3, entitle “participation of employees in decisions making” had better score. The results of Yaghoubi and Javadi^23^ study in private and governmental hospitals were close to the results of the present study. In line with the accreditation program, the hospitals had some HP plan for staff. However, structural reforms are necessary to create a health supportive environment for staff. Employee motivation and success of HP programs in the hospitals might be increased by employee participation in plans and creating healthy working environment.

 The lack of clear assessment guideline was one of the limits of this study. Despite this limita­tion, this study provided key information on the status of HP in the educational hospitals in Isfahan city. Further studies in the other hospitals and cities are recommended because of rare performed investigations.

 The present finding can notify policy makers and managers of areas of HP activities which hospitals are doing well in and areas where they need to improve. These results can also help developing HPH initiatives.

## Conclusion

 Though some of the activities and HP standards were running out in the studied hospitals, but in line with the accreditation program, these activities tended to come under the banner of the quality management program, rather than being described as HP activities. Many of the hospitals in Iran already have or are in the process of acquiring various quality certifications. So it could be a good opportunity in the hospitals for shift to the HPH policy.

## Ethical approval

 This study received approval from the ethics committee of Isfahan University of Medical Sciences with project number: 393761, December 2014. All officials and assessment teams staff in the Treatment Administration and hospitals gave their oral consents for participation in this research. All in­formation of the hospitals was kept confidential and they were allowed to discontinue participation at any stage of the research. Time and date of the assessment were set with evaluation teams in hospitals.

## Competing interests

 The authors declare that there is no conflict of interests.

## Acknowledgments

 This article is resulted from PhD dissertation in health education and promotion at Isfahan University of Medical Sciences in 2015 (Project No: 393761). The authors express their gratitude to hospital staff for their cooperation, and, to Treatment Administration and Department of Research and Technology of Isfahan University of Medical Science for their support. The authors declare that there is no conflict of interests.
